# Erosion–Corrosion Behavior of TiC-Based Cermets with Fe-Ni Binder in Artificial Seawater

**DOI:** 10.3390/ma19194074

**Published:** 2026-09-23

**Authors:** Bin Wu, Rong Zhang, Zhixing Guo, Zhi Xie, Xiangyu Song, Xiang Xia, Guang Xian, Tianen Yang, Linzhi Xie, Feichi Chen, Yunyi Zhu, Mei Yang

**Affiliations:** 1 College of Materials and Chemistry & Chemical Engineering, Chengdu University of Technology, Chengdu 610059, China; 13981286308@163.com (B.W.); 15883276708@163.com (R.Z.); 13308215872@163.com (L.X.);; 2 School of Mechanical Engineering, Sichuan University, Chengdu 610065, China; xz23sacd@163.com (Z.X.); yangtze86@163.com (T.Y.); 3 Zhejiang Zhenxing Petrochemical Machinery Co., Ltd., Wenzhou 325200, China

**Keywords:** TiC, cermets, Fe content, erosion–corrosion resistance, artificial seawater

## Abstract

TiC-based cermets are promising candidates for marine wear-resistant components; however, achieving a balance between mechanical performance, erosion–corrosion resistance, and binder cost remains a major challenge. In this work, TiC-xFe-(15-x)Ni (x = 0, 5, 10 and 15 wt.%) cermets were fabricated by vacuum liquid-phase sintering to clarify the influence of Fe/Ni binder composition on the microstructure, mechanical properties, electrochemical behavior, and erosion–corrosion resistance in SiO_2_-containing artificial seawater. The results indicate that the Fe content has a significant impact on liquid-phase sintering behavior, thereby affecting the densification of cemented carbides. The combination of TiC-5Fe-10Ni achieved the highest relative density (98.89%), Vickers hardness (1618 HV), and fracture toughness (10.26 MPa·m^1/2^), while also exhibiting the lowest corrosion current density in electrochemical corrosion tests. In contrast, TiC-10Fe-5Ni cemented carbide showed the lowest cumulative mass loss in long-term erosion–corrosion tests. Conversely, excessive Fe addition leads to suboptimal performance. The performance improvement is mainly attributed to densification and reduced residual porosity achieved through the optimization of liquid-phase sintering, which provides an effective strategy for designing cost-effective TiC-based composites with balanced mechanical properties and long-term corrosion and erosion resistance.

## 1. Introduction

Cermets are widely used in cutting tools, mining equipment, and wear-resistant components owing to their excellent hardness, wear resistance, and high-temperature stability [1,2]. Conventional WC-based cermets, consisting of WC hard phase and Co binder, have dominated industrial applications for decades due to their outstanding mechanical properties [3]. However, the increasing demand for strategic tungsten resources and the high cost of cobalt have stimulated the development of alternative hard materials and binder systems. Consequently, TiC-based cermets have attracted increasing attention due to their high hardness ulated the development of alternative hard materials and binder systems [4,5]. Although WC-Co cermets exhibit excellent mechanical performance, the scarcity of tungsten resources and the volatility of cobalt prices have become important limitations for their large-scale and sustainable application. Moreover, cobalt exhibits relatively poor corrosion resistance in aggressive environments, which may accelerate binder dissolution and deteriorate long-term service reliability [6,7,8]. Therefore, developing tungsten-free hard materials and low-cost binder systems has attracted increasing research interest. In addition to conventional wear-resistant applications, cemented carbide components are increasingly required to operate under harsh marine environments, where seawater erosion, chloride-induced corrosion, and mechanical impact can occur simultaneously. Therefore, improving the erosion–corrosion resistance of cermet materials has become an important consideration for their application in marine engineering equipment [9,10,11].

TiC-based cermets have been considered as promising alternatives to WC-based cermets because of their lower density, higher melting temperature, excellent chemical stability, and comparable hardness [12,13]. Compared with conventional WC-based materials, TiC-based cermets exhibit superior oxidation resistance and good chemical inertness, making them attractive candidates for applications requiring high wear resistance under severe service conditions. In marine environments, metal–ceramic components are frequently subjected to the combined effects of seawater erosion and electrochemical corrosion, which can accelerate surface degradation and reduce service reliability [14]. Therefore, improving the erosion–corrosion resistance of cermet components is essential for their application in marine engineering equipment. Owing to their high hardness, excellent wear resistance, good chemical stability, and relatively low susceptibility to corrosion, TiC-based cermets are considered promising materials for manufacturing wear-resistant and corrosion-resistant components used in marine environments. Among various binder systems, Ni has been widely employed as the metallic binder due to its excellent wettability with TiC and superior corrosion resistance. Exner et al. [1] systematically investigated the microstructure evolution of TiC-Ni cermets and demonstrated that the dissolution and precipitation behavior of TiC during liquid-phase sintering contributed to the formation of a characteristic core–rim structure, which improved the mechanical stability of the material. Ettmayer et al. [12] further reported that TiC-Ni cermets exhibit excellent wear resistance and thermal stability, making them suitable for cutting and wear-resistant applications. Compared with WC-Co or WC-Ni cermets, TiC-Ni cermets have the advantage of lower cost, as well as lower density, and superior high-temperature hardness, wear resistance, and oxidation resistance. Despite these advantages, TiC-Ni cermets still suffer from insufficient fracture toughness compared with WC-Co systems, mainly due to the limited strengthening capability of the Ni binder. Therefore, optimization of the metallic binder composition has become a key strategy for further improving the comprehensive properties of TiC-based cermets [15,16,17,18].

To further enhance the comprehensive properties of TiC-based cermets, alloyed Ni binders have been extensively studied. For example, Lei et al. [19] used the vacuum solid-droplet method to wet the Ag-Cu-Zn alloy on TiC-Ni cermet and investigated the interfacial microstructure. At the Ag-Cu-Zn/TiC-Ni cermet interface, after heating at 810 °C for 10 min, a continuous (Cu,Ni) solid-solution layer was formed, thereby affecting the performance of the cermet. Liu et al. [20] added Ni_3_Ti to TiC-based cermets and studied its sintering behavior, microstructure, and mechanical properties. The results indicate that the addition of Ni_3_Ti improves the fracture toughness of the cermets. In addition, Cr-containing Ni binders have been reported to improve oxidation and corrosion resistance by promoting the formation of protective chromium oxide films, while Co-containing binders can enhance toughness through solid-solution strengthening. Although Ni-based alloy binders have achieved significant improvements in mechanical properties and environmental stability, the relatively high cost of Ni-containing materials limits their application in large-scale engineering fields. Therefore, developing low-cost binder systems with comparable mechanical performance and improved service reliability has become an important research direction [21,22,23,24].

With the development of alloy design theory, Fe-Ni solid-solution systems have been widely investigated as promising binder phases for cermets. Carbonyl iron powder (hereinafter referred to as Fe) is a micron-sized elemental iron powder obtained by the carbonylation reaction of sponge iron with carbon monoxide to produce liquid iron pentacarbonyl Fe (CO)_5_, which is then subjected to gas-phase thermal decomposition. Compared with Ni, Fe has advantages such as low cost, high hardness, and strong affinity for carbides, making it an ideal material to replace or partially replace nickel-based binders. Fe and Ni can form a stable face-centered cubic solid solution under high-temperature sintering, which introduces significant lattice distortion and enhances dislocation resistance. This solid-solution strengthening mechanism has been widely applied in designing high-performance metallic binders for ceramic–metal composites. And in TiC-based cermets, Fe-Ni binders have mainly been investigated from the perspective of mechanical property optimization. Several researchers reported that Fe-Ni alloy binders promote the formation of homogeneous metallic phases, refine TiC grains, and improve hardness and fracture toughness. For instance, Wang et al. [25] investigated TiC-Fe/Ni cermets and demonstrated that appropriate Fe content could enhance densification and mechanical properties through solid-solution strengthening and microstructural refinement. Beyond TiC-based systems, Fe-Ni binders have also been widely explored in WC-based cermets, especially regarding corrosion behavior. Research by Human and Exner showed that the corrosion resistance of cermets is strongly affected by binder composition, and Ni-containing binders generally exhibit improved corrosion stability compared with Co or Fe binders. Furthermore, studies on WC-FeNi cermets revealed that Fe-Ni alloy binders can provide a balance between mechanical strength and corrosion resistance by forming more stable passive films in corrosive environments [26,27,28,29].

However, previous studies on WC-FeNi and TiC-Ni cermets have primarily focused on improving densification and mechanical properties through optimization of the metallic binder. Although several investigations have reported enhanced hardness or fracture toughness by adjusting the Fe/Ni ratio, comparatively few studies have examined how binder composition influences the coupled interaction between erosion–corrosion and electrochemical corrosion in particle-containing marine environments. Consequently, the relationship among binder composition, microstructural evolution and long-term erosion–corrosion performance remains insufficiently understood. The present work aims to bridge this gap by systematically investigating the influence of Fe addition on phase evolution, densification, mechanical properties, electrochemical corrosion and erosion–corrosion behavior of TiC-based cermets. To address these research gaps and meet the practical performance requirements of TiC-based cermets, in this work, TiC-Fe/Ni cermets with different Fe content were fabricated by vacuum powder metallurgy technology, and the total metallic binder content was fixed at 15 wt.% while Fe progressively replaced Ni from 0 to 15 wt.%. This composition range was selected to investigate the evolution from a pure Ni binder to a Fe-rich Fe-Ni binder while maintaining sufficient liquid-phase-assisted sintering. According to the Fe-Ni binary phase diagram, Fe and Ni exhibit complete solid solubility in the face-centered cubic phase at elevated temperatures, facilitating the formation of substitutional (Fe,Ni) solid solutions during sintering. However, excessive Fe addition may reduce liquid-phase formation because Fe has a higher melting temperature than Ni. Therefore, the selected composition range provides a suitable balance between binder modification and sintering feasibility. The effects of Fe addition on phase evolution, microstructure development, densification behavior, mechanical properties, and erosion–corrosion resistance were systematically investigated. The formation mechanism of the (Fe,Ni) solid solution, strengthening mechanism, and corrosion degradation process were analyzed to establish the relationship between composition, microstructure, and performance. In addition, in practical applications such as marine engineering, components like pump impellers, valve seats, and slurry transport parts often face the combined effects of solid particle erosion and chloride corrosion. Although conventional TiC-based hard composites exhibit excellent wear resistance, the degradation of the metallic binder phase often limits their service life in harsh marine environments. Therefore, this study aims to optimize the ratio of Fe/Ni binder phases in order to simultaneously enhance mechanical reliability and erosion–corrosion performance for potential marine wear-resistant applications [30].

## 2. Experimental Section

### 2.1. Preparation of TiC-Based Cermets

Commercially available TiC (Zhuzhou China), Fe (Jinchuan Jinchang China), and Ni (Zhuzhou China) powders were used as raw materials. Characteristics of these powders are listed in Table 1, and the nominal composition of the cermets is tabulated in Table 2. The raw material powder was ball-milled in a planetary ball mill (DM-4L, Nanjing, China) using ethanol as the medium for 12 h. The ball-to-powder mass ratio was 5:1, and the milling speed was 300 rpm. Apart from ethanol, no other process control agents were used. After the milling, the slurry was extracted intermittently, and then dried at 80 °C for 2 h. Then, a molding agent, prepared by mixing gasoline and SD rubber (cermet molding Agent) at a ratio of 1:1, was added to the powder at approximately 0.8 mL/g, and then dried at 80 °C. The powder was sieved through an 80-mesh sieve, and then rounded green samples were compacted using a uniaxial press machine (769YP-24B, Hunan, China). The samples were sintered in a vacuum sintering furnace (ZT-40-21Y, Shanghai, China) at 1440 °C for 1 h, and then cooled to room temperature After sintering, the actual density, radial shrinkage, volumetric shrinkage, and theoretical density of the samples are determined by measuring their diameter, height, and mass. The actual density is the average of three samples (n = 3), while the other data represent typical results [12].

### 2.2. Electrochemical Testing

Figure 1a is a schematic diagram of a three-electrode system, consisting of a working electrode, a reference electrode (saturated calomel), and an auxiliary electrode (platinum sheet) (CHI660e, Shanghai, China). The electrolyte is a 3.5 wt.% NaCl solution. The three electrodes form two circuits, as shown in Figure 1b. P represents the polarization power supply, and mA and V represent the ammeter and voltmeter, respectively. The sample was embedded in nonconductive resin, and then the exposed surface of the sample was sequentially polished with 60-, 800-, 1600-, and 2400-grit polishing compounds, followed by cleaning in ethanol using an ultrasonic bath. One end of a copper wire was flattened and connected to the sample with conductive copper adhesive, then embedded in a mold with crystal resin (epoxy resin). The prepared working electrode is shown in Figure 1c, with a testing area of approximately 1 cm^2^. Then corrosion tests are conducted using an electrochemical workstation. The potential range for the potentiodynamic polarization test was −0.6 V to 0.4 V, with a scan rate of 0.001 V/s. The obtained electrochemical measurement results are representative results obtained under the same experimental conditions [31,32].

### 2.3. Erosion–Corrosion Test

Then, various compounds were added according to ASTM D1141-98 (2008) [33], and artificial seawater was prepared. Silica (SiO_2_) particles of grit size 60–80 mesh were used as abrasives, and 10 wt.% was added into artificial seawater. The four different cermet samples are placed together in the erosion–corrosion equipment illustrated in Figure 2. The erosion–corrosion equipment was rotating at 400 rpm. Erosion–corrosion time was selected to be 60 days for all samples. And the samples are cylindrical with an exposed area of approximately 4.7 cm^2^, experiments were conducted at four different positions and orientations of the samples within the container, the electrolyte volume was 1000 mL, the temperature was room temperature. Each sample was weighed before and after each day’s erosion–corrosion test to an accuracy of 0.0001 g. The electrolyte and/or particles were replaced every ten days according to the sample weight. The results obtained are representative under the same experimental conditions. The removed samples were ultrasonically cleaned and dried, and their positions were exchanged when placed back, which differs from the actual conditions of erosion under real circumstances. The erosion–corrosion resistance of the material is characterized by mass loss per unit area, and the formula is shown in Equation (1).

(1) $$P_{w}=\frac{∆W}{A}$$

where P_w_ is mass loss per unit area in g/cm^2^, △W is mass loss before and after erosion–corrosion in g, and A is surface area of samples in cm^2^.

### 2.4. Characterization

The phase composition analysis was investigated by X-ray diffractometer (XRD; DX-2700, Dandong Fangyuan Instrument Co., China) with CuKα radiation (wavelength, 0.15406 nm), operated at 40 kV, 30 mA, 2θ at 15–80°. The microstructures and morphology were determined by scanning electron microscope (SEM, PHENOM PROX, China). The density is measured by the Archimedes drainage method. Vickers hardness and fracture toughness are measured by indentation method of the Vickers hardness tester (HV_30_, HMAS-D1000SZ, China).

Hardness is essentially an empirical value and, for many materials, can be correlated with tensile strength. It also reflects wear resistance and ductility. In this experiment, the indentation method based on Vickers hardness testing was used. A 136° diamond indenter applied an experimental force of F = 294.8 N, with a dwell time of 15 s. A rhombic indentation was produced on the sample surface. By measuring the diagonal lengths of the indentation and performing calculations, the Vickers hardness value HV30 was obtained. To ensure more accurate experimental results, three measurements were taken on the polished surface of each sample, and the average was taken as the Vickers hardness value of that sample. During the Vickers hardness measurement, the four edges of the rhombic indentation will each propagate a crack. The lengths of the four cracks, L1, L2, L3, and L4, were measured and substituted into formula 2 to calculate the fracture toughness of the sample. Figure 3 is a schematic diagram of the indentation method based on Vickers hardness testing:

(2) $$K_{IC}=0.15\sqrt{\frac{Hv30}{\sum Li}}$$

where K_IC_ is the fracture toughness of the sample in MPa·m^1/2^; HV30 is the Vickers hardness value; and Σ Li is the total length from the indentation tip to the crack tip in mm.

The fracture toughness results are calculated based on representative hardness results.

## 3. Results and Discussion

### 3.1. Phase Analysis

Figure 4 shows the XRD patterns of TiC-based cermets with different Fe content. As shown, all samples are predominantly composed of TiC (PDF#65-8804) as the main crystalline phase, and the characteristic diffraction peak positions match the standard card closely, indicating that the TiC ceramic phase maintains a stable face-centered cubic crystal structure under different metallic ratios, with no significant lattice distortion or formation of secondary phases. In the single-metal addition systems, the TiC-15Fe sample exhibited a pure Fe phase (PDF#85-1410), with characteristic diffraction peaks corresponding to the (110), (200), and (211) planes; the TiC-15Ni sample showed a pure Ni phase (PDF#70-0989), with characteristic peaks corresponding to the (111), (200), and (220) planes. In the Fe-Ni co-added systems (TiC-5Fe-10Ni and TiC-10Fe-5Ni), the characteristic peaks of elemental Fe and Ni gradually disappear, and instead a new diffraction peak corresponding to the (Fe,Ni) solid-solution phase (PDF#47-1417) emerges [34,35,36]. This indicates that during the vacuum sintering process, Fe and Ni underwent sufficient mutual diffusion, forming a stable face-centered cubic solid solution. The formation of the (Fe,Ni) phase can be attributed to the similar atomic radii (Fe: 0.126 nm, Ni: 0.124 nm), identical crystal structure at high temperature (FCC (face-centered cubic)), and comparable electronegativity, which satisfy the Hume-Rothery solid solubility rules, thus promoting complete or near-complete miscibility between Fe and Ni. Further mechanism analysis indicates that in the TiC-Fe/Ni system, Fe acts as a toughening phase that effectively mitigates the intrinsic brittleness of the ceramic phase, while the formed (Fe,Ni) solid solution significantly affects residual stress distribution, lattice distortion, and microhardness. The phase composition of the metallic bonding phase has a significant impact on the interfacial bonding and mechanical properties of TiC-based composites. Compared with pure iron or nickel, the formation of an (Fe,Ni) solid solution not only ensures wetting and filling capabilities during the liquid-phase sintering process, but also, under the presence of TiC, the addition of iron can reduce the liquid-phase formation temperature of the TiC-FeNi system, allowing the liquid phase to form earlier and thereby promoting liquid-phase sintering and densification. Consequently, even in the early stages of sintering, a continuous and penetrating metallic liquid phase can be formed, further enhancing material densification and optimizing interfacial bonding strength. Moreover, by adjusting the iron content, the physicochemical properties of the solid solution can also be modified, providing a theoretical basis for the functional design of the composites [13,37].

### 3.2. Microstructure

The microstructure of cermets is usually composed of ceramic hard phase and metal bonded phase. Figure 5 shows the SEM/BSE (back-scattered electron) microtopography of TiC-xFe-(15-x)Ni cermets. As can be seen from the figure, all cermet specimens contain a dark hard phase and a light bonded phase, with a typical “core–edge” structure. The microstructure contains three types of grains, namely the weak core-gray edge structure, the black core-gray edge structure, and the white core-gray edge structure. Among them, the core of the gray edge structure of the black core is black, and the gray edge is dominated by gray, because the atoms with lower atomic numbers produce darker images under scanning electron microscopy, so the light element content of the black core is richer than that of the gray edge. The evolution of the microstructure strongly depends on the binder composition, which influences particle rearrangement, liquid-phase transport, and densification during sintering [13].

The TiC-15Ni specimen (Figure 5a) contains relatively coarse hard-phase particles, discontinuous binder distribution and a noticeable amount of residual pores, indicating incomplete densification. In contrast, the addition of 5 wt.% and 10 wt.% Fe results in a more homogeneous microstructure with fewer pores and a more continuous metallic network (Figure 5b,c). The improved densification originates from the enhanced sintering behavior of the Fe-Ni binder rather than from an intrinsic grain-refining effect of Fe. During sintering at 1440 °C, the Ni-rich binder approaches its melting temperature and provides efficient liquid-phase transport. The dissolution of Fe into Ni promotes the formation of an FCC (Fe,Ni) solid solution, facilitating atomic diffusion, particle rearrangement and capillary-driven pore elimination. Consequently, the TiC-5Fe-10Ni and TiC-10Fe-5Ni specimens exhibit a denser microstructure with significantly reduced residual porosity. However, when the Fe content increases to 15 wt.% (Figure 5d), the amount of Ni becomes insufficient to sustain effective liquid-phase sintering because pure Fe remains completely solid at the sintering temperature. As a result, pore elimination becomes less effective, leading to increased porosity and microstructural heterogeneity. It should be noted that the apparent TiC grain size observed after sintering does not directly correspond to the initial TiC powder size (0.89 μm). During liquid-phase sintering, partial dissolution of TiC into the Ni-rich binder is followed by reprecipitation onto existing carbide particles. Simultaneously, Ostwald ripening and limited particle coalescence occur, producing the characteristic core–rim structure of TiC-based cermets. Therefore, the hard-phase size measured from SEM images represents the final microstructure after dissolution–reprecipitation rather than simple solid-state growth of the original TiC particles [38,39].

Overall, the microstructural evolution is primarily governed by the liquid-phase sintering behavior of the Fe-Ni binder. Appropriate Fe addition improves densification through enhanced particle rearrangement and pore filling, whereas excessive Fe suppresses liquid-phase transport because of the high melting temperature of Fe, resulting in inferior densification and increased residual porosity [12].

### 3.3. Properties

Figure 6 presents the variation of theoretical density, practical density, relative density, Vickers hardness, and fracture toughness of TiC-Fe/Ni cermets as a function of Fe content. The theoretical density shows a slight decreasing trend with increasing Fe content due to the lower atomic mass and density of Fe compared with Ni. However, the practical density exhibits a non-linear evolution, first increasing and then decreasing with Fe addition. The highest densification is achieved at intermediate Fe content. Specifically, the relative density increases from 97.99% (0 wt.% Fe) to a maximum value of 98.89% at 5 wt.% Fe, followed by a slight decrease to 98.28% (10 wt.% Fe) and a more pronounced drop to 95.78% at 15 wt.% Fe, indicating that excessive Fe addition deteriorates the densification behavior [40].

The Vickers hardness exhibits a similar trend, increasing first and then decreasing with Fe content. The hardness reaches a maximum value of approximately 1618 HV at 5 wt.% Fe, indicating improved resistance to plastic deformation. This enhancement can be attributed to the combined effects of improved densification, solid-solution strengthening of the (Fe,Ni) binder phase, and grain refinement of TiC particles, which collectively increase the resistance to dislocation motion and localized plastic deformation. Similarly, the fracture toughness shows a peak value at 5 wt.% Fe (approximately 10.26 MPa·m^1^/^2^), followed by a decline at higher Fe content [41,42]. The improvement in fracture toughness is mainly associated with crack deflection, crack bridging, and pull-out mechanisms induced by the uniformly distributed TiC hard phase and the ductile (Fe,Ni) metallic binder. The continuous metallic phase effectively blunts crack tips and dissipates fracture energy, thereby enhancing the damage tolerance of the composite [43,44,45,46].

The observed mechanical behavior can be explained by the synergistic strengthening mechanisms operating in the TiC-Fe/Ni system. First, solid-solution strengthening occurs due to the formation of a homogeneous FCC (Fe,Ni) phase, which introduces lattice distortion and impedes dislocation motion. Second, grain refinement strengthening follows the Hall–Petch relationship, where reduced TiC grain size increases grain boundary density and enhances strength. Third, densification strengthening arises from reduced porosity and improved interfacial bonding, which minimizes stress concentration sites. Finally, crack propagation is effectively hindered by TiC particles through crack deflection and bridging mechanisms, contributing to enhanced fracture toughness. However, when Fe content increases to 15 wt.%, the microstructure becomes less stable due to insufficient liquid-phase continuity and partial particle agglomeration, leading to increased porosity, reduced densification, and weakened grain refinement effects. As a result, both hardness and fracture toughness decrease significantly [47,48].

Overall, the optimal mechanical performance is achieved at 5 wt.% Fe, where the synergistic effects of high densification, solid-solution strengthening, and grain refinement are maximized. This component provides an optimal balance between hardness and fracture toughness, indicating that precise control of different Fe content is crucial for tailoring the mechanical properties of TiC-based cermets.

### 3.4. Electrochemical Test

In order to study the effect of Fe content on the electrochemical corrosion performance of TiC-based cermets, the corrosion resistance of the cermet samples was tested in a 3.5% NaCl solution at room temperature. Figure 7 shows the potentiodynamic polarization curves (Tafel curves) of TiC-based cermets with different Fe contents. From the figures, it can be seen that the Tafel curves of the cermets exhibit a generally consistent trend, with the transition point between the cathodic and anodic regions corresponding to the corrosion potential (Ecorr). This point can also be determined from the intersection of the extrapolated cathodic and anodic Tafel lines, giving the corrosion current density (Icorr). It is clearly evident from the figures that the TiC-15Ni, TiC-5Fe-10Ni, and TiC-10Fe-5Ni cermet samples show relatively high and similar corrosion potentials, indicating that these three samples have a lower tendency to corrode. In contrast, the TiC-15Fe cermet sample exhibits the lowest corrosion potential, indicating a higher tendency for corrosion. Furthermore, for the TiC-5Fe-10Ni sample, after cathodic polarization is completed, the corrosion current increases gradually with the rising corrosion potential and then enters a relatively stable region, indicating that the sample surface possesses relatively good inherent corrosion resistance. For the remaining three samples, after cathodic polarization is completed, the corrosion current initially increases and then decreases as the corrosion potential continues to rise. A turning point appears near a corrosion potential of 0.347 V_VS._SCE (the potential is equivalent to that of a saturated calomel electrode), These results are consistent with the relative density measurements, where TiC-5Fe-10Ni exhibits the highest densification, whereas TiC-15Fe contains the highest residual porosity. The improved electrochemical performance of the TiC-5Fe-10Ni cermet is considered to be primarily associated with its enhanced microstructural integrity. Appropriate Fe addition promotes liquid-phase-assisted densification of the Fe-Ni binder, resulting in reduced residual porosity and improved continuity of the metallic binder network. The denser microstructure effectively restricts electrolyte penetration into the interior of the cermet and reduces the number of preferential corrosion sites, thereby lowering the corrosion current density. Conversely, the higher porosity of the TiC-15Fe specimen facilitates electrolyte ingress and localized dissolution of the metallic binder, leading to accelerated corrosion. The polarization behavior of the Ni-containing cermets is also consistent with improved surface electrochemical protection. However, the composition, thickness and stability of any surface passive layer were not directly characterized in the present study. Therefore, the polarization results alone cannot demonstrate the formation of a stable or dense passive film. Any possible contribution of surface passivation should be regarded as a plausible interpretation of the electrochemical response rather than definitive evidence. Consequently, the superior corrosion resistance of the TiC-5Fe-10Ni cermet is more reasonably attributed to its improved densification and reduced residual porosity, while differences in passivation behavior, if present, may provide an additional contribution.

Overall, the polarization results indicate that optimization of the Fe/Ni binder composition significantly influences the electrochemical behavior of TiC-based cermets. The electrochemical response observed in the present study is closely correlated with the microstructural characteristics established during liquid-phase sintering, suggesting that enhanced densification and reduced pore connectivity are the principal factors responsible for the improved corrosion resistance [17,49].

In order to study the corrosion resistance of the surface of TiC-based cermets during electrochemical corrosion in 3.5 wt.% NaCl solution, EIS plots (Electrochemical Impedance Spectroscopy, including the Nyquist and Bode plots) of TiC-xFe-(15-x)Ni cermets were measured and are shown in Figure 8. Typically, in the high-frequency region of the Nyquist plot, a single relatively regular capacitive arc can be observed, indicating that the corrosion process is mainly controlled by charge transfer rather than mass diffusion. The magnitude of the impedance is reflected by the radius of the capacitive arc, which can be used to compare the corrosion resistance of different samples. Generally, the larger the radius of the capacitive arc, the better the corrosion resistance of the sample. As shown in Figure 8a, all samples exhibit the typical characteristic of a single capacitive arc, and the arc diameter shows significant differences with the change in Fe content; the radius of the arc for the TiC-5Fe-10Ni sample is significantly larger than that of the other three samples, indicating the highest polarization resistance, the strongest charge transfer resistance, and the best corrosion resistance, which is consistent with the results obtained from the polarization curves. In contrast, the TiC-15Fe sample has the smallest arc radius, the lowest polarization resistance, and the poorest corrosion resistance, which is inconsistent with the results obtained from the polarization curves [5,15,17].

Figure 8b shows the Bode phase angle plot of TiC-xFe-(15-x)Ni cermets. Such Bode plots depicting the phase angle in relation to frequency can generally indicate the number of electrochemical processes, serving as a means to study corrosion processes and mechanisms. From the figure, it can be seen that TiC-15Fe exhibits a single peak, indicating only one time constant during the electrochemical reaction, whereas the other three cermet samples display two peaks, indicating the presence of two time constants. Figure 8b shows that the TiC-15Ni sample has the highest phase angle peak and the widest plateau; in contrast, the TiC-15Fe sample shows a rapid decay in the phase angle, approaching 0° in the high-frequency region. Besides, Figure 8c shows the TiC-10Fe-5Ni cermet and TiC-10Fe-5Ni cermet exhibit the higher impedance modulus over the measured frequency range, while the TiC-15Fe specimen shows the lowest impedance values. These results indicate that optimization of the Fe/Ni binder composition significantly influences the electrochemical response of the cermets. Combined with the density measurements and microstructural observations, the improved electrochemical behavior is more reasonably associated with the enhanced densification and reduced residual porosity obtained during liquid-phase sintering. A denser microstructure restricts electrolyte penetration into the binder phase and reduces the number of preferential corrosion sites, thereby improving the overall corrosion resistance. And these results can be seen from Table 3. Although the electrochemical responses of the Ni-containing cermets are consistent with improved surface passivation behavior, the composition, thickness and stability of the passive layer were not directly characterized in the present study. Therefore, the EIS results alone do not provide sufficient evidence to conclude that a stable or dense passive film was formed. Accordingly, the electrochemical behavior observed in this work should be interpreted as being possibly associated with differences in surface passivation together with the improved microstructural integrity resulting from enhanced densification. In the present study, the latter is considered to be the dominant factor governing the corrosion performance. Figure 8d shows a schematic diagram of electrochemical impedance circuit commonly used in previous studies to describe the electrochemical response of cermets in chloride-containing environments. The circuit is included only as a conceptual illustration of the possible electrochemical processes contributing to the impedance response and was not used for equivalent-circuit fitting in the present work. Therefore, no quantitative fitting parameters or physical interpretations of individual circuit elements (e.g., Rs, R_1_, R_2_, CPE_1_ and CPE_2_) are presented. The discussion of the EIS results is intentionally limited to qualitative comparison of the experimental Nyquist and Bode plots, which is fully supported by the available experimental evidence.

Overall, the EIS results demonstrate that the TiC-10Fe-5Ni cermet possesses the highest electrochemical impedance among the investigated compositions. This behavior is consistent with its higher relative density, lower residual porosity and superior corrosion resistance obtained from the polarization and erosion-corrosion tests. Therefore, the enhanced corrosion resistance is primarily attributed to the improved microstructural integrity established during liquid-phase sintering, while any contribution of surface passivation should be regarded as a possible auxiliary factor requiring further verification by direct surface characterization techniques, such as XPS or TEM [30,31,32,49].

### 3.5. Erosion–Corrosion Test

Figure 9 shows the surface morphologies of the TiC-xFe-(15-x)Ni cermets after 60 days of erosion–corrosion in artificial seawater. Compared with the as-sintered surfaces, all specimens exhibit typical features of binder dissolution, carbide exposure and particle pull-out, indicating that material degradation results from the combined action of electrochemical corrosion and erosion–corrosion. However, the extent of surface damage varies significantly with binder composition, reflecting the differences in the microstructural integrity established during liquid-phase sintering. For the TiC-15Ni specimen (Figure 9a), only a limited number of isolated pits and local binder removal are observed, while most TiC particles remain well anchored in the metallic matrix. The relatively intact surface morphology indicates that the continuous binder network effectively maintains the carbide/matrix bonding during erosion–corrosion. In contrast, the TiC-15Fe specimen (Figure 9d) exhibits extensive interconnected pits, severe binder dissolution and large-scale TiC particle pull-out. Numerous cavities and interfacial cracks are visible, suggesting that electrolyte penetration preferentially occurred through residual pores and weak interfacial regions, leading to progressive degradation of the metallic binder and subsequent loss of carbide support. The TiC-5Fe-10Ni and TiC-10Fe-5Ni cermets (Figure 9b,c) exhibit intermediate surface damage. Compared with the TiC-15Fe specimen, both cermets contain fewer interconnected pits and retain a more continuous metallic skeleton after erosion–corrosion. Among them, TiC-10Fe-5Ni exhibits the least carbide pull-out and the smallest damaged area, indicating superior structural stability during long-term exposure. This behavior is consistent with the higher relative density and lower residual porosity obtained after sintering. The improved erosion–corrosion resistance is therefore mainly associated with enhanced densification, which reduces pore connectivity, restricts electrolyte ingress and improves the interfacial bonding between the metallic binder and TiC particles. Similar relationships between reduced porosity and improved corrosion resistance have been widely reported for liquid-phase-sintered powder metallurgy materials.

It should be noted that although the polarization and impedance results suggest improved passivation behavior for the Ni-containing cermets, the present work does not directly characterize the chemical composition or structure of the passive layer. Therefore, the observed differences in surface morphology cannot be conclusively attributed to differences in passive-film stability. Instead, the SEM observations demonstrate more directly that microstructural integrity, particularly reduced porosity and improved binder continuity, plays the dominant role in suppressing electrolyte penetration and delaying binder dissolution under erosion–corrosion conditions.

The erosion–corrosion behavior observed in the present study is consistent with the degradation mechanisms reported for cermets and metallic materials exposed to particle-containing saline environments. Previous electrochemical studies have demonstrated that erosion–corrosion is governed by the synergistic interaction between erosion–corrosion and electrochemical corrosion rather than by either process acting independently. Under repeated solid-particle impingement, the surface layer is continuously damaged, exposing fresh metallic binder to the electrolyte and initiating localized anodic dissolution. Subsequent repassivation may occur on the newly exposed surface; however, repeated particle impacts repeatedly disrupt this process, producing a cyclic depassivation–repassivation process that accelerates material degradation. This synergistic interaction has been recognized as one of the dominant mechanisms controlling erosion–corrosion in slurry environments [50]. Similar degradation behavior has also been reported for Ti (C,N)-based cermets in SiO_2_-containing artificial seawater. Guo et al. [51] reported that erosion–corrosion resistance is closely related to the integrity of the metallic binder network and the resistance of the binder phase to localized dissolution. Once the metallic binder is preferentially removed, the surrounding hard particles gradually lose mechanical support and are eventually detached under continuous particle impacts, resulting in accelerated material loss. The present SEM observations (Figure 9) exhibit the same characteristic features, including preferential binder dissolution, carbide exposure, interfacial debonding and TiC particle pull-out, indicating that binder degradation dominates the subsequent erosion–corrosion process rather than direct fracture of the TiC phase. Combining the present electrochemical results with previous erosion–corrosion models suggests that the superior performance of the TiC-10Fe-5Ni cermet results primarily from its improved microstructural integrity. The higher densification obtained after liquid-phase sintering reduces pore connectivity and delays electrolyte penetration into the binder phase. Consequently, localized dissolution of the metallic binder and subsequent particle detachment are effectively suppressed during repeated SiO_2_ particle impacts. Although the polarization and impedance results indicate improved passivation behavior for the Ni-containing cermets, the chemistry and stability of the passive layer were not directly characterized in the present study. Therefore, the passive layer is more appropriately regarded as a secondary factor that may influence the electrochemical response, whereas the dominant mechanism responsible for the improved erosion–corrosion resistance is the enhanced densification and interfacial stability achieved through optimization of the Fe/Ni binder composition.

Figure 10 presents the cumulative mass loss of the TiC-xFe-(15-x)Ni cermets during 60 days of erosion–corrosion. All specimens exhibit an initially rapid mass-loss stage followed by a relatively slower degradation stage. The initial increase in mass loss is mainly associated with the preferential dissolution of exposed metallic binder and the removal of surface asperities before a relatively stable corrosion process is established. As erosion–corrosion proceeds, the degradation rate becomes increasingly dependent on the microstructural integrity of the cermets rather than the initial electrochemical response. The TiC-15Fe specimen exhibits the highest cumulative mass loss throughout the entire test, whereas TiC-10Fe-5Ni shows the lowest value after 60 days. The higher residual porosity in TiC-15Fe provides interconnected pathways for electrolyte penetration, facilitating localized corrosion of the binder phase and accelerating interfacial degradation under repeated particle impacts. Once the metallic binder is dissolved, the TiC particles lose mechanical support and are readily detached from the surface, resulting in continuous mass loss. Conversely, the denser microstructure of TiC-10Fe-5Ni effectively suppresses electrolyte penetration, delays binder dissolution and maintains stronger carbide/matrix bonding, thereby significantly reducing material removal. These observations agree well with previous studies showing that porosity is one of the dominant factors controlling the corrosion behavior of powder metallurgy materials. Compared with TiC-10Fe-5Ni, the TiC-5Fe-10Ni specimen exhibits a slightly higher mass loss during the later stage of testing. In the original manuscript, this behavior was attributed to insufficient passive-film toughness. However, such an interpretation is not directly supported by the present experimental evidence because no chemical or structural characterization of the passive layer was performed. A more reasonable explanation is that the remaining residual pores and local interfacial defects gradually became preferential pathways for electrolyte penetration during prolonged erosion–corrosion. Under repeated impacts of SiO_2_ particles, localized binder dissolution and interfacial debonding accumulated progressively, leading to accelerated TiC particle pull-out during the later stage of exposure. Although differences in passivation behavior may also contribute to the electrochemical response, their individual contribution cannot be distinguished from the present results and requires further surface characterization, such as XPS or TEM analyses, for verification. The evolution of cumulative mass loss shown in Figure 10 further supports the synergistic erosion–corrosion mechanism. During the initial stage, preferential dissolution of the exposed metallic binder dominates the degradation process. As erosion–corrosion proceeds, repeated SiO_2_ particle impacts continuously remove corrosion products and newly formed surface films, exposing fresh metallic surfaces to the electrolyte. The repeated depassivation–repassivation cycle promotes continuous electrochemical dissolution of the binder phase, while mechanical impacts progressively weaken the carbide/binder interfaces. Once sufficient interfacial degradation has accumulated, TiC particles are detached from the surface, leading to accelerated material removal. Similar degradation sequences have been reported for both metallic alloys and cermets subjected to slurry erosion–corrosion, demonstrating that the total material loss cannot be explained solely by electrochemical corrosion or erosion–corrosion, but instead results from their synergistic interaction.

Overall, the erosion–corrosion degradation of the TiC-Fe/Ni cermets follows a progressive sequence of electrolyte penetration through residual pores → preferential dissolution of the metallic binder → interfacial debonding → TiC particle pull-out → continuous material loss under repeated erosion [50]. Appropriate Fe addition improves the erosion–corrosion resistance primarily by promoting liquid-phase-assisted densification of the Fe-Ni binder, thereby reducing residual porosity and enhancing interfacial integrity. The electrochemical measurements further suggest improved passivation behavior in the Ni-containing cermets; however, the chemistry and stability of the passive layer were not directly characterized in the present study. Therefore, its contribution should be regarded as a possible auxiliary factor rather than the primary mechanism responsible for the observed erosion–corrosion behavior.

## 4. Conclusions

TiC-Fe/Ni cermets (TiC-xFe-(15-x)Ni, x = 0, 5, 10, 15 wt.%) were prepared using the vacuum powder metallurgy method. The phase composition, microstructural evolution, mechanical properties, and slurry erosion behavior of the alloy were systematically investigated. The main conclusions are summarized as follows.

(1) The Fe/Ni content significantly influences the liquid-phase sintering behavior and consequently determines the microstructural evolution of the cermets. Appropriate Fe addition promotes the formation of a Fe–Ni solid solution, enhancing particle rearrangement and pore elimination during sintering. As a result, the TiC-5Fe-10Ni and TiC-10Fe-5Ni cermets exhibit denser and more homogeneous microstructures than the single-binder systems. In contrast, excessive Fe addition (15 wt.%) suppresses effective liquid-phase transport because of the higher melting temperature of Fe, resulting in increased residual porosity and microstructural heterogeneity.

(2) The mechanical properties are closely correlated with the densification behavior of the cermets. The TiC-5Fe-10Ni specimen exhibits the highest relative density (98.89%), Vickers hardness (1618 HV) and fracture toughness (10.26 MPa·m^1/2^), indicating the optimum balance between strength and toughness. The improved mechanical performance is primarily attributed to reduced residual porosity, enhanced binder continuity and stronger carbide–binder interfacial bonding produced during liquid-phase sintering, whereas excessive Fe addition deteriorates these properties owing to insufficient densification.

(3) The electrochemical behavior is also strongly affected by the Fe content. The TiC-5Fe-10Ni cermet exhibits the lowest corrosion current density, indicating the best electrochemical corrosion resistance. The improved electrochemical response is mainly associated with its dense microstructure, which suppresses electrolyte penetration and reduces preferential dissolution of the metallic binder. Although the electrochemical measurements are consistent with improved surface passivation behavior in the Ni-containing cermets, the passive layer was not directly characterized in the present study. Therefore, the superior corrosion resistance is primarily attributed to enhanced densification and reduced residual porosity rather than to passive-film effects.

(4) During long-term erosion–corrosion in SiO_2_-containing artificial seawater, the TiC-10Fe-5Ni cermet exhibits the lowest cumulative mass loss, demonstrating the best erosion–corrosion resistance. This behavior is attributed to the combined effects of a dense microstructure, improved interfacial integrity and greater resistance to binder degradation and carbide pull-out during repeated particle impact.

Overall, optimization of the Fe/Ni binder composition improved the overall performance of TiC-based cermets primarily by enhancing liquid-phase sintering and densification. The present results demonstrate that microstructural integrity, particularly relative density and residual porosity, is the dominant factor controlling the mechanical properties and erosion–corrosion resistance, while the influence of passivation requires further investigation through direct surface characterization techniques.

## Figures and Tables

**Figure 1:**
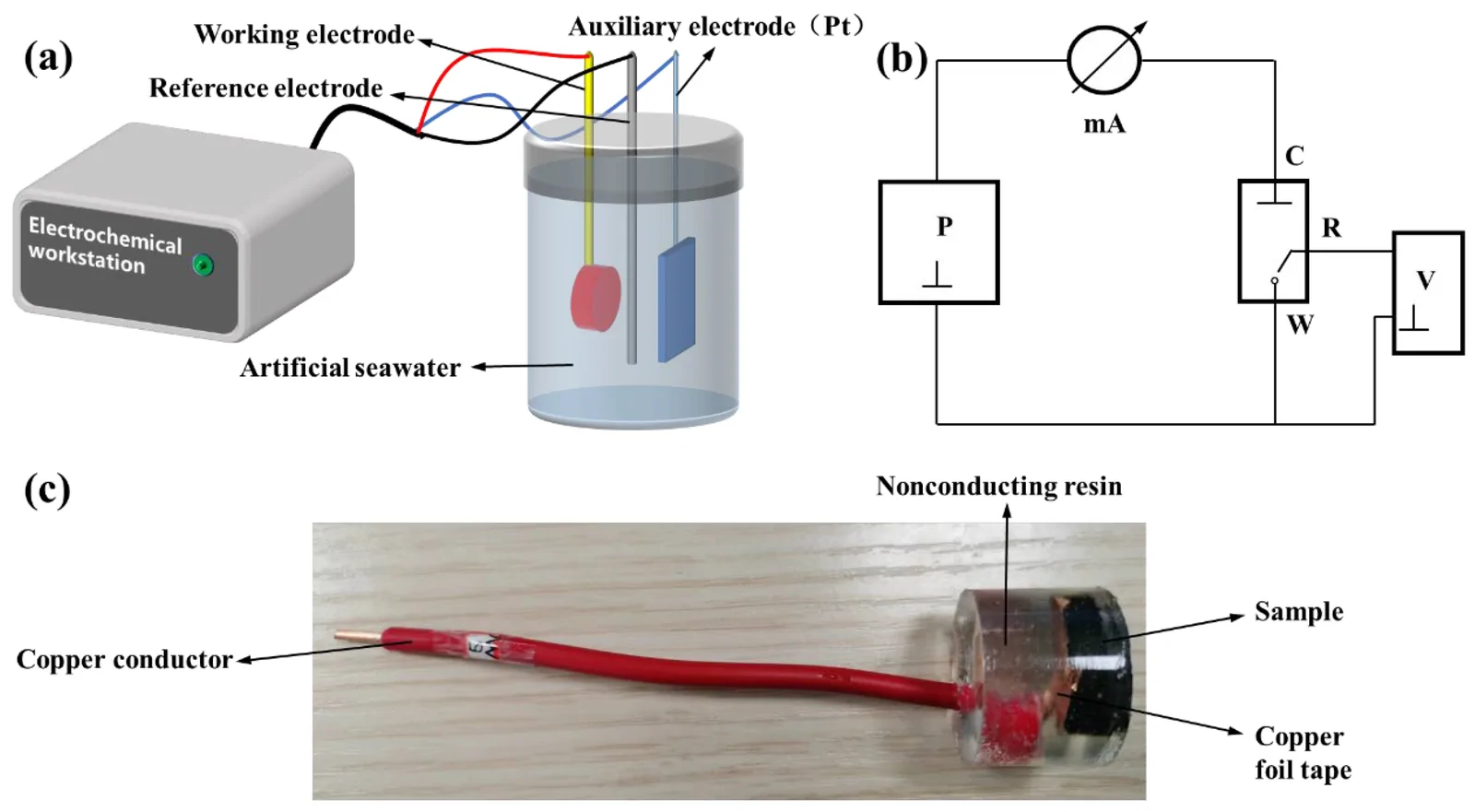
Schematic diagram of electrochemical test: (**a**) three-electrode system structure; (**b**) circuit diagram; (**c**) working electrode.

**Figure 2:**
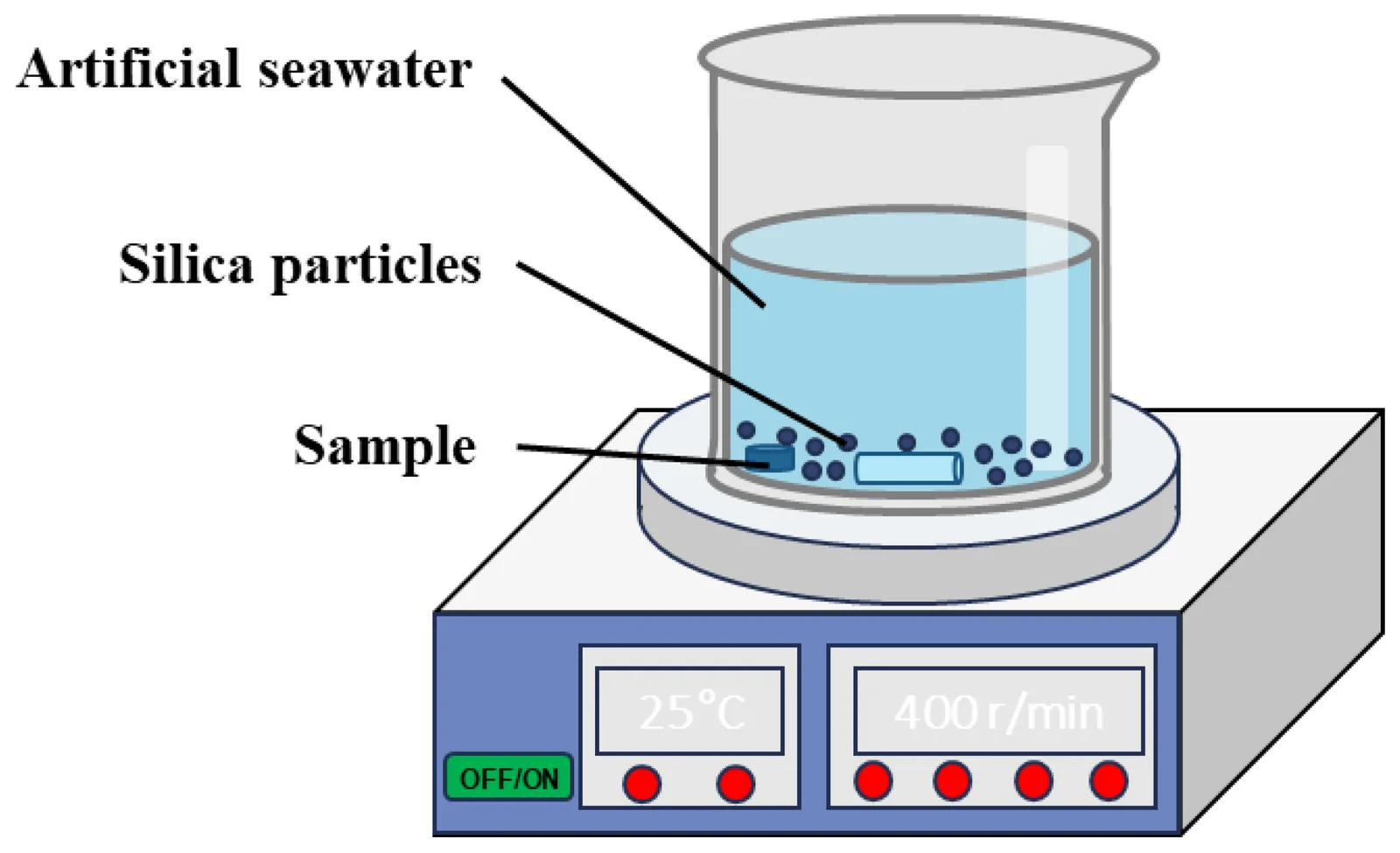
Schematic diagram of erosion–corrosion experiment.

**Figure 3:**
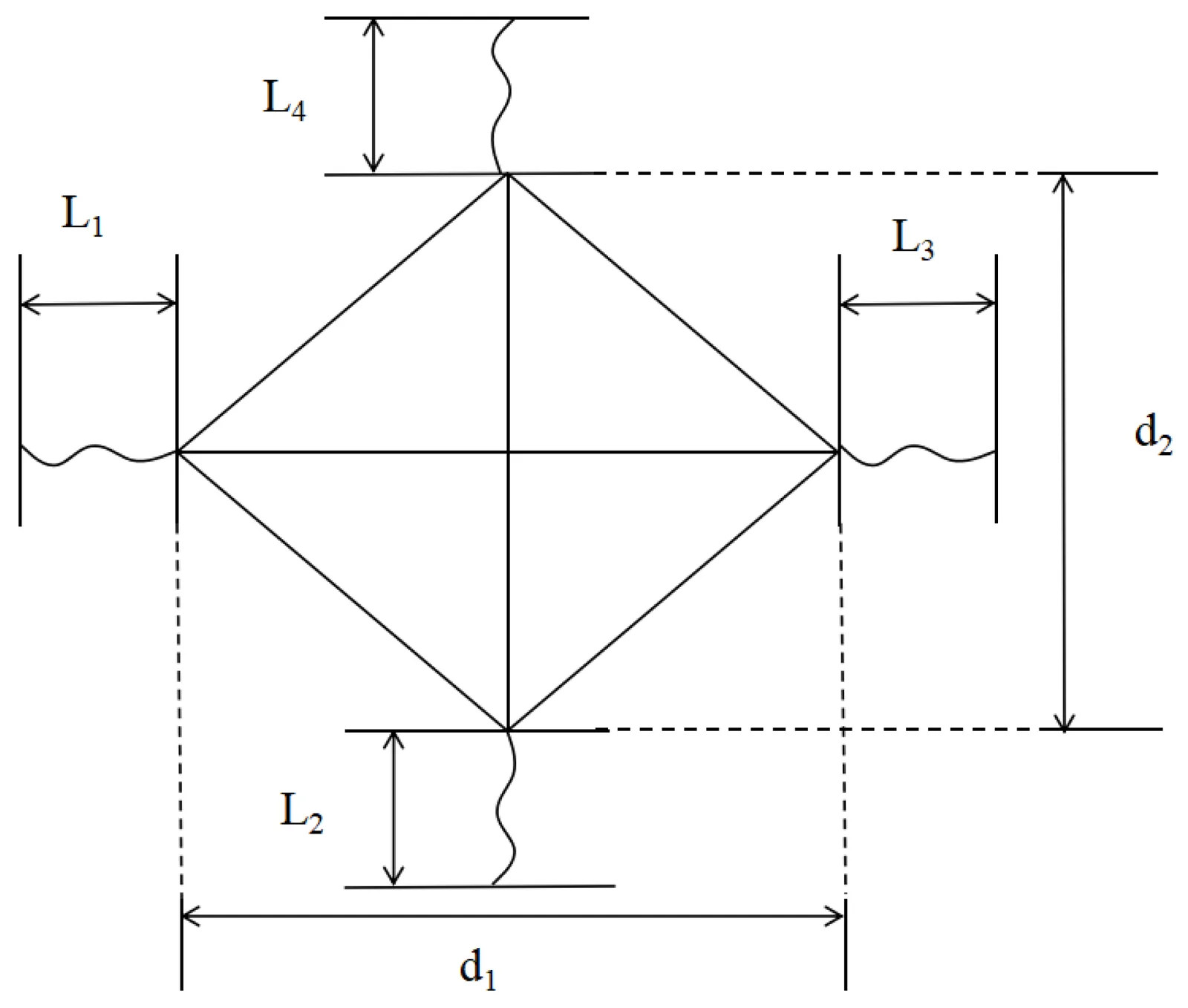
Schematic of fracture toughness test by indentation method.

**Figure 4:**
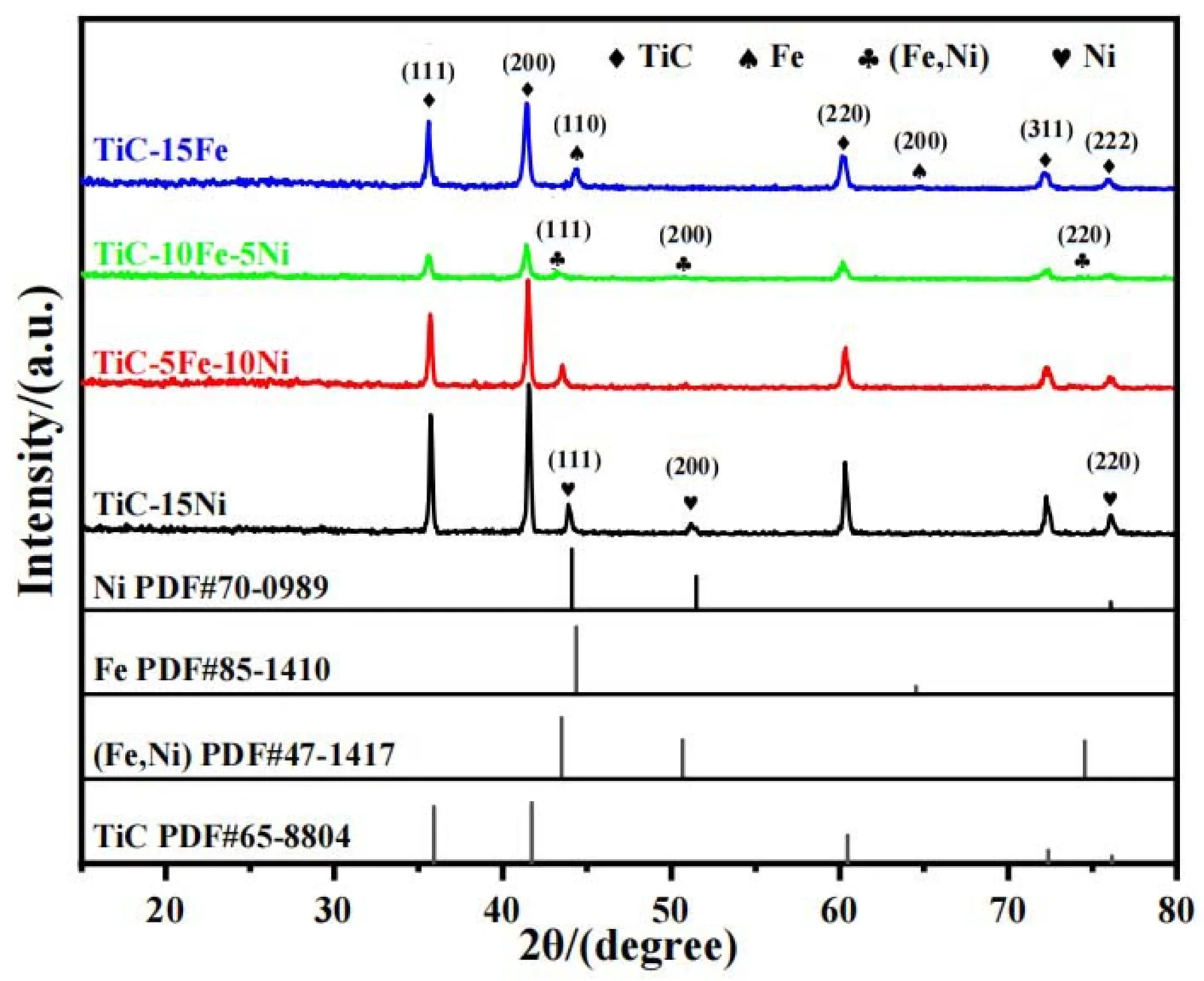
XRD patterns of TiC-xFe-(15-x)Ni (x = 0, 5, 10, 15) cermets.

**Figure 5:**
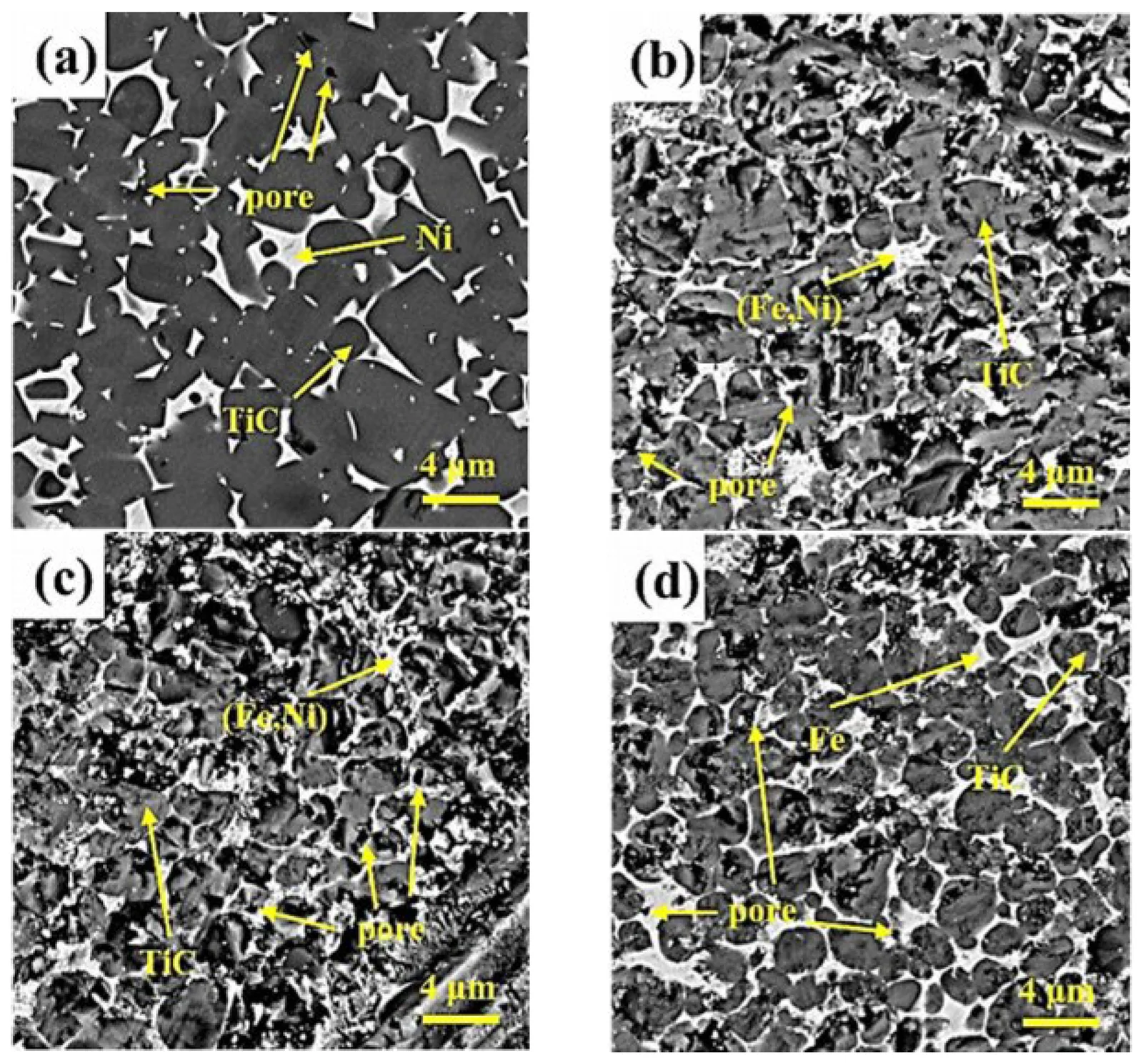
SEM/BSE micrographs of the TiC-xFe-(15-x)Ni (x = 0, 5, 10, 15) cermets: (**a**) x = 0 wt.%, (**b**) x = 5 wt.%, (**c**) x = 10 wt.%, (**d**) x = 15 wt.%.

**Figure 6:**
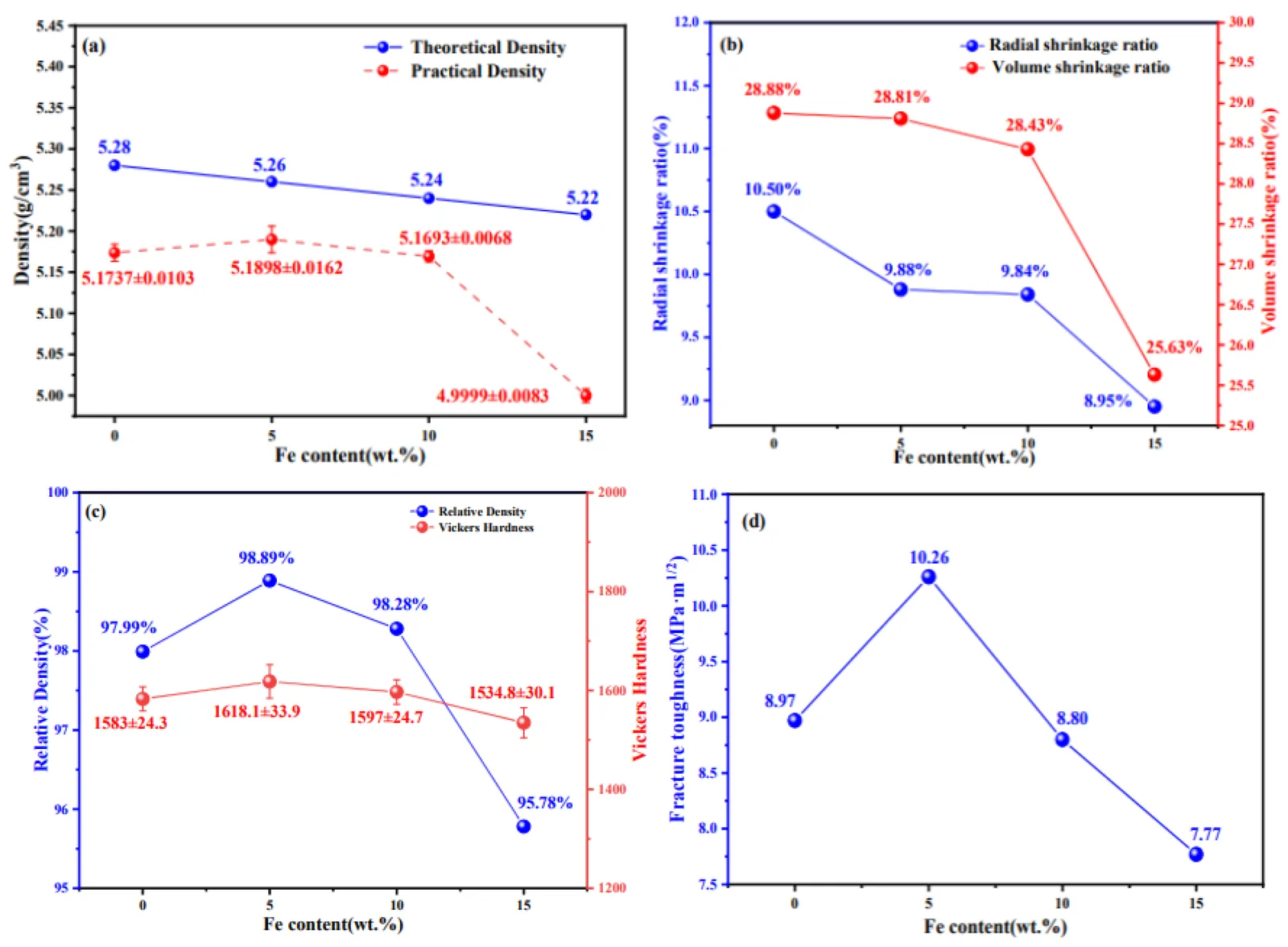
Mechanical properties of TiC-xFe-(15-x)Ni (x = 0, 5, 10, 15) cermets: (**a**) density; (**b**) shrinkage ratio; (**c**) relative density and Vickers hardness; (**d**) fracture toughness. (The practical density and Vickers hardness represent the results of three measurements averaged, with error bars indicating the standard deviation (n = 3). The others are selected representative results).

**Figure 7:**
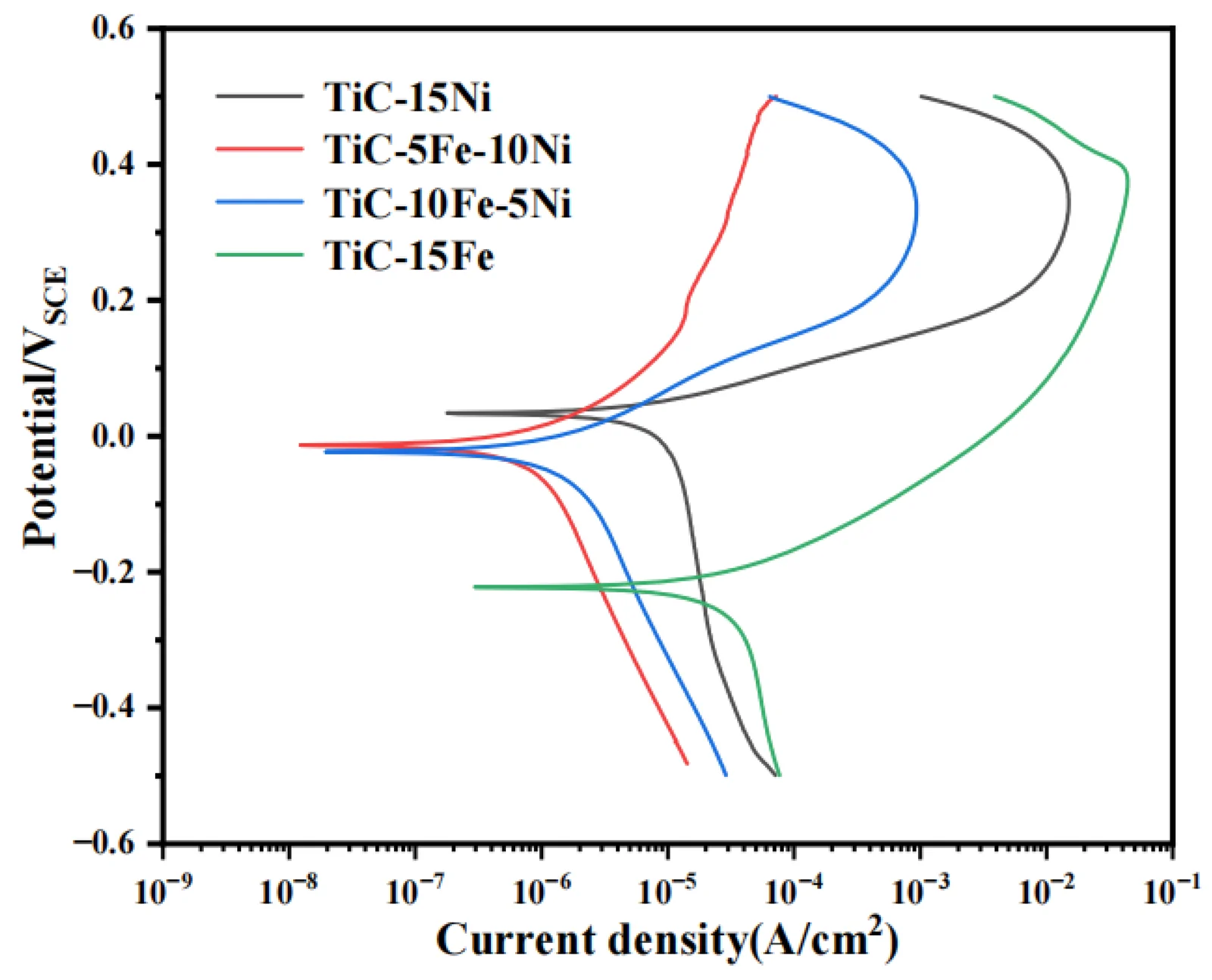
Potentiodynamic polarization curves of TiC-based cermets with different Fe content.

**Figure 8:**
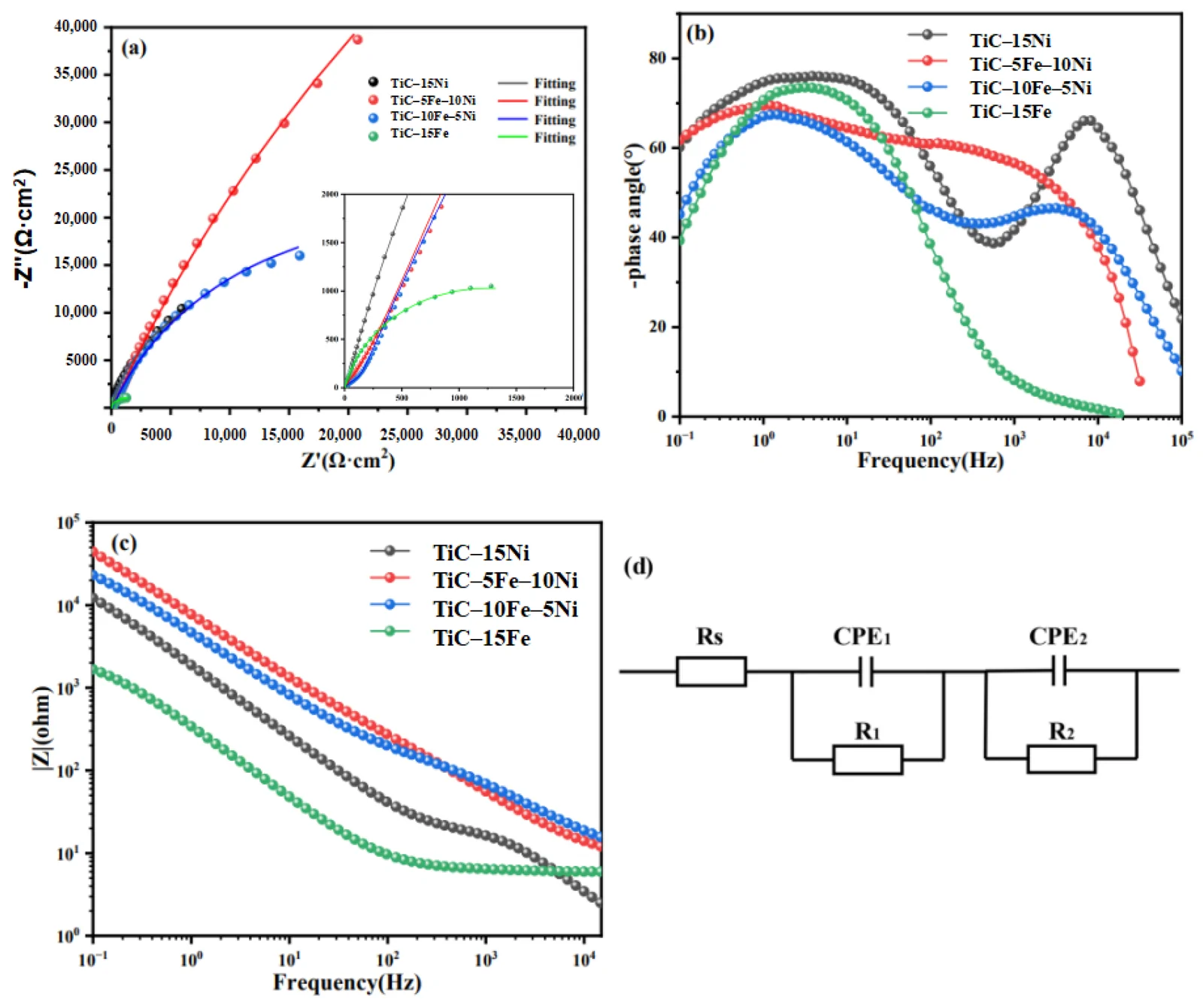
EIS plots of TiC-xFe-(15-x)Ni (x = 0, 5, 10, 15) cermets in 3.5 wt.% NaCl aqueous solution: (**a**) Nyquist plots, inset was the partial enlarged plots; (**b**) Bode plots of phase angle versus frequency; (**c**) Bode plot of |Z| versus frequency; (**d**) Schematic Diagram of Electrochemical Impedance Circuit. The obtained electrochemical measurement results are representative results obtained under the same experimental conditions.

**Figure 9:**
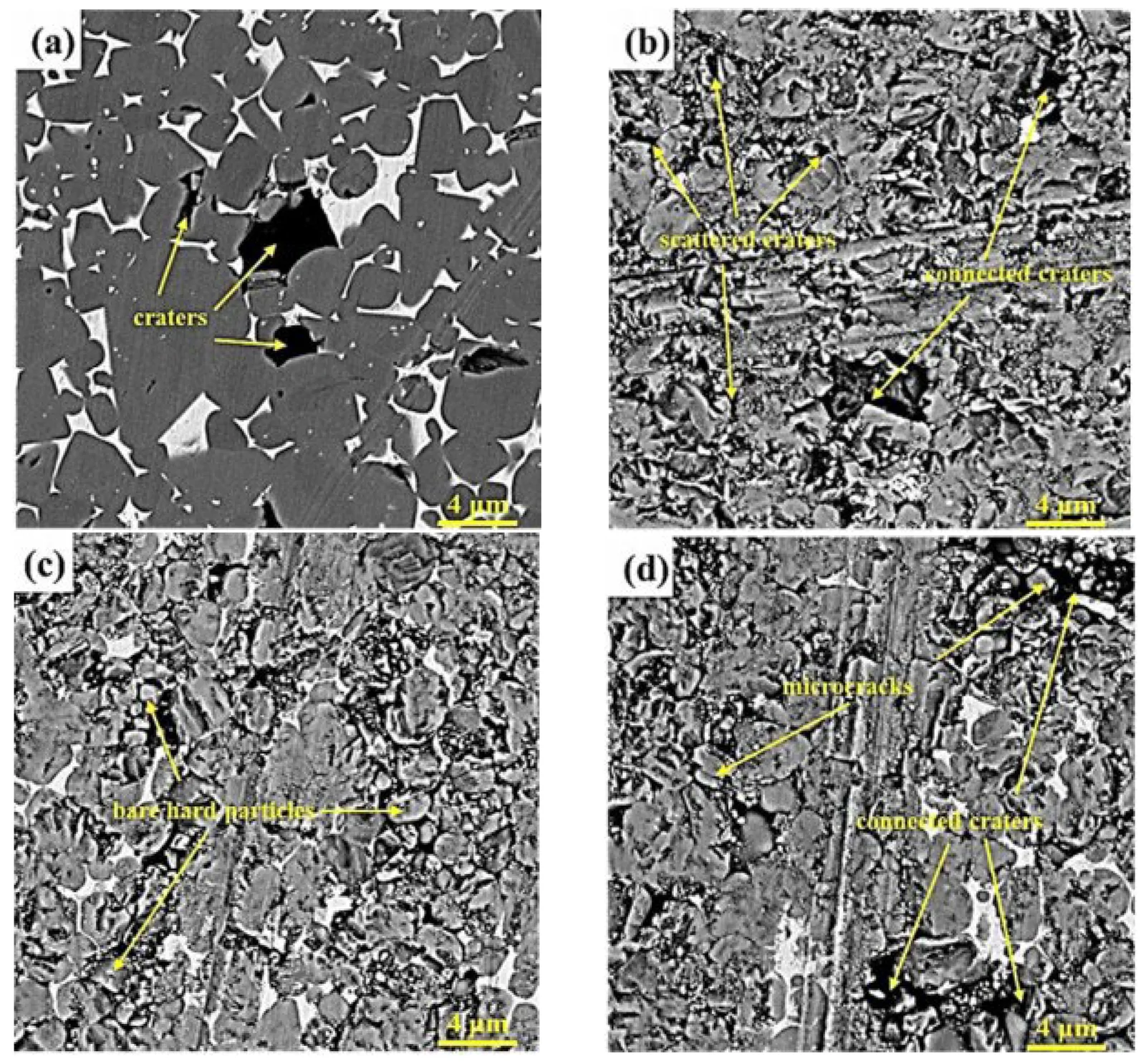
Erosion–corrosion surface micrographs of the TiC-xFe-(15-x)Ni (x = 0, 5, 10, 15) cermets: (**a**) x = 0 wt.%, (**b**) x = 5 wt.%, (**c**) x = 10 wt.%, (**d**) x = 15 wt.%.

**Figure 10:**
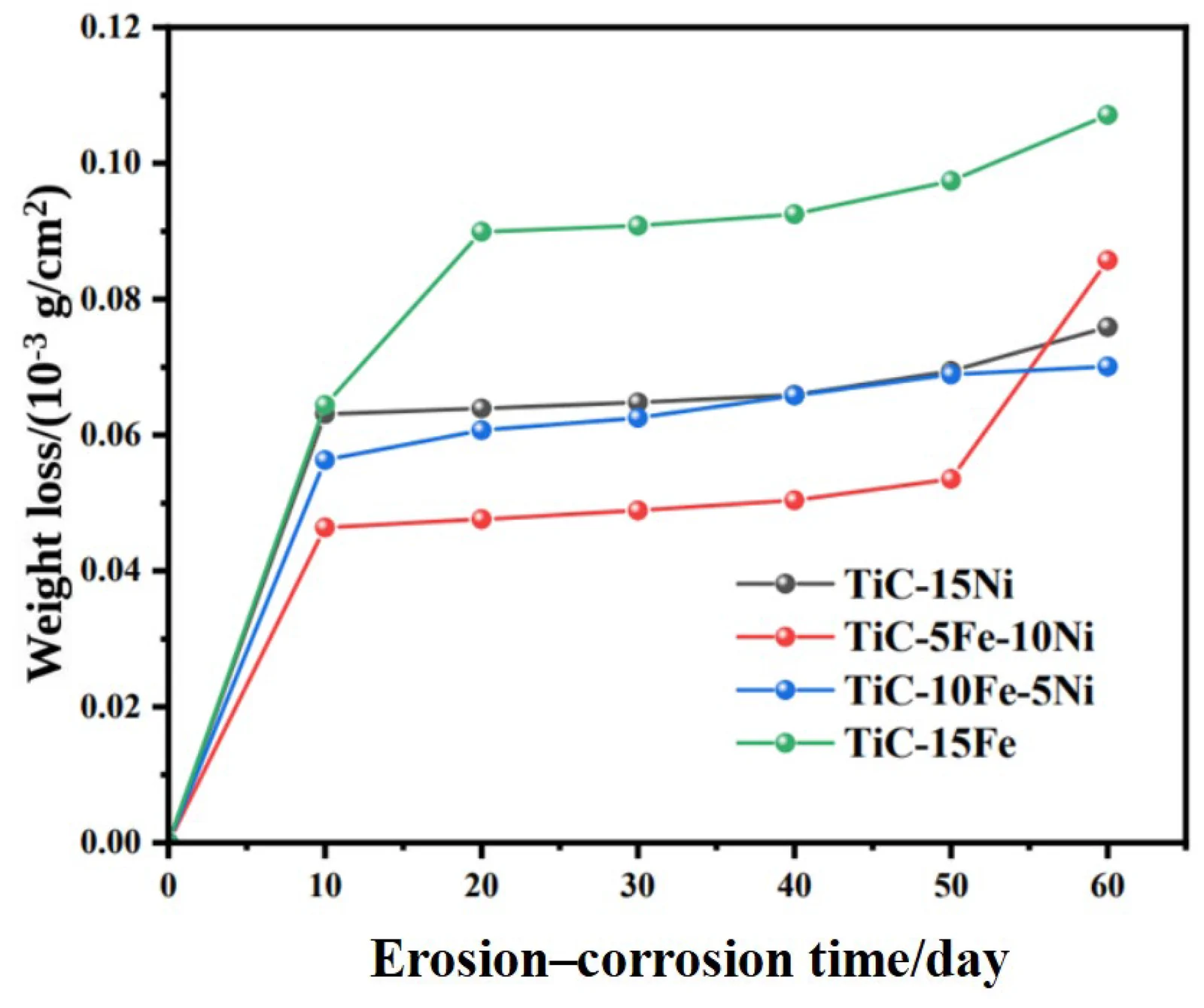
Erosion–corrosion weight loss curves of TiC based cermets with different Fe content. (The weightless measurement results shown were obtained under the same experimental conditions and are representative results).

**Table 1:** Characteristics of the raw powders.

Powder	Particle Size (μm)	Purity (wt%)	Manufacturer
TiC	0.89	>99.0	Zhuzhou Cemented Carbide Group Corp., LTD. of China
Ni	2.25	>99.7	Zhuzhou Cemented Carbide Group Corp., LTD. of China
Fe	1.41	≥99.5	Jinchuan Group Co., LTD. of China

**Table 2:** Composition design of the experimental materials.

Cermets	TiC (wt.%)	Fe (wt.%)	Ni (wt.%)
TiC-15Ni	85	0	15
TiC-5Fe-10Ni	85	5	10
TiC-10Fe-5Ni	85	10	5
TiC-15Fe	85	15	0

**Table 3:** The relationship between the density of TiC cermets with different compositions and the data or parameters.

Sample	Relative Density	Ecorr (V_VS._SCE)	Icorr (μA/cm^2^)	IE (%)
TiC-15Ni	97.99	0.034	7.664	-
TiC-5Fe-10Ni	98.89	0.021	1.182	84.58
TiC-10Fe-5Ni	98.28	−0.022	1.547	79.81
TiC-15Fe	95.78	−0.022	3.344	56.37

## Data Availability

The original contributions presented in this study are included in the article. Further inquiries can be directed toward the corresponding authors.
